# Integration of a soft dielectric composite into a cantilever beam for mechanical energy harvesting, comparison between capacitive and triboelectric transducers

**DOI:** 10.1038/s41598-020-77581-2

**Published:** 2020-11-26

**Authors:** Mickaël Pruvost, Wilbert J. Smit, Cécile Monteux, Pablo Del Corro, Isabelle Dufour, Cédric Ayela, Philippe Poulin, Annie Colin

**Affiliations:** 1 MIE Team, Chimie Biologie Et Innovation, ESPCI Paris, PSL University, CNRS, 75005 Paris, France; 2 Sciences Et Ingénierie de La Matière Molle, ESPCI Paris, PSL University, CNRS, Sorbonne Université, 75005 Paris, France; 3 Laboratoire IMS, CNRS, Université de Bordeaux, 33600 Pessac, France; 4 Centre de Recherche Paul Pascal, CNRS, Université de Bordeaux, 33600 Pessac, France

**Keywords:** Energy harvesting, Devices for energy harvesting

## Abstract

Flexible dielectrics that harvest mechanical energy via electrostatic effects are excellent candidates as power sources for wearable electronics or autonomous sensors. The integration of a soft dielectric composite (polydimethylsiloxane PDMS-carbon black CB) into two mechanical energy harvesters is here presented. Both are based on a similar cantilever beam but work on different harvesting principles: variable capacitor and triboelectricity. We show that without an external bias the triboelectric beam harvests a net density power of 0.3 $\upmu \mathrm{W}/{\mathrm{cm}}^{2}$ under a sinusoidal acceleration of 3.9*g* at 40 Hz. In a variable capacitor configuration, a bias of 0.15 $\mathrm{V}/\upmu \mathrm{m}$ is required to get the same energy harvesting performance under the same working conditions. As variable capacitors’ harvesting performance are quadratically dependent on the applied bias, increasing the bias allows the system to harvest energy much more efficiently than the triboelectric one. The present results make CB/PDMS composites promising for autonomous portable multifunctional systems and intelligent sensors.

## Introduction

A large amount of untapped energy sources surrounds us. They derive from human activities or come from ambient energies such as chemical, thermal, radiant and mechanical energies. Contrary to batteries, they are not time-dependent and can power low-power electronics (ie. nW, mW) for long periods. After the radiant sources (sun, RF, etc.), mechanical energy (vibrations, wind, rain, etc.) is the second main environmental source of energy. It can be found almost anywhere: from ocean waves to industrial facilities. Considering its ubiquity, vibrational energy is of particular interest to be converted into electricity. Different transducers (electromagnetic, piezoelectric or capacitive transducers) are studied for decades, their operating principle and limitations are well known^1^. Among these recognized technologies, triboelectric transducers (TENG) enjoy a growing interest, in particular thanks to Pr. ZL Wang's pioneering work^2^. Their performances are notable; for example, Wang et al. succeeded in harvesting 150 ${\upmu \mathrm{W}}/{\mathrm{cm}}^{2}$ (instantaneous peak power) using a broadband TENG at a frequency of 36 Hz^3^. From the shape of the current intensity, one may assume that the average power is at least five or ten times lower than the peak power, leading to an estimated power from 15 to 75 ${\upmu \mathrm{W}}/{\mathrm{cm}}^{2}$. By integrating a mechanical spring-based system in a TENG, Wang et al. succeeded in obtaining an average power density of 0.2 $\upmu \mathrm{W}/{\mathrm{cm}}^{2}$ for a frequency of 3.5 Hz and an acceleration of 1*g*^4^. Based on electrostatic effects, TENG can be compared to capacitive transducers. Indeed, in both systems charge density is a key factor. But charges are injected via different mechanisms. For triboelectric systems, electrical charges come from a physical contact between the two dissimilar dielectrics facing with each other^5^, whereas in capacitive harvesting systems they come from a DC-voltage bias^6^. This is one of the main drawbacks of triboelectricity as charges densities are very sensitive to moisture and can also disappear due to air breakdown^7–9^. On the contrary, electrical charges in capacitive harvesting systems are less sensitive to external conditions. But their accumulation is limited by the leak resistance of the capacitor. Another distinction is the theoretical level of understanding of the two systems. Indeed, capacitive transducers are based on a well-established capacitor principle, whereas the microscopic mechanisms of triboelectricity are still debated. For example, after centuries of research, it is still not clear whether electrons, ions or even bulk material transfer is responsible for the observed charging^10^. The research for developing new TENG’s is still in progress, especially to improve the harvested power and to establish a clearer theory on the subject.

In addition to this new field of research, capacitive transducers have been the subject of renewed interest^11^, in particular thanks to recent progresses on dielectric elastomers which have a higher permittivity than air^12–15^. Typically, dielectric elastomers are inserted between compliant electrodes to form a multilayered electrical capacitor with deformation dependent capacitance. Several structures of capacitive transducers have been studied and applied to different sources of mechanical energy from wave energy^16^ to human motion^17^. Largely employed for piezoelectric generators, the design and realization of cantilever beams have been less investigated in the context of electrostatic transducers (capacitive and triboelectric transducers). However, the structure of cantilever beam is generally simple and well suited to produce large strain in particular at the mechanical resonant frequency. An efficient way to design a capacitive transducer as a beam is to insert a flexible dielectric material between two electrodes, and to clamp the so created capacitance to a shaker. When the shaker is subjected to vibrations, the cantilever beam oscillates thereby varying the capacitance formed by the soft dielectric and the two electrodes (parallel-plate capacitor). At resonant frequency, the beam oscillates with a maximal amplitude and generates large variations of capacitances. This induces oscillating displacements of electrons when the dielectric is polarized, and therefore the production of electrical energy along a cycle.

Based on the same cantilever architecture, a triboelectric energy harvester can be easily implemented by inserting two dielectrics facing each other. Indeed, triboelectricity occurs when two dissimilar materials get charged by contacting and separating them (dielectric-to-dielectric attached-electrode parallel-plate contact-mode^5,18^). It offers a charge-pump to drive electrons through an external resistance load, in which current flows back and forth between the electrodes as alternating current. Contrary to a capacitive transducer, a triboelectric transducer does not require any external polarization.

Here, we study a new soft dielectric^19,20^, an emulsion based PDMS/carbon black composite, in the framework of electrostatic beam transducers. For two harvesting configurations (triboelectric and capacitive harvesters) the recovery powers are discussed. Considering losses of the systems, we show that the capacitive transducer beam provides a positive energy balance. We measured a net power density of 0.15 $\upmu \mathrm{W}/{\mathrm{cm}}^{2}$ for an acceleration of 5.4*g* and a frequency of 40 Hz under a bias voltage of 50 mV/$\mathrm{\mu m}$. These performances are lower than the ones obtained by a triboelectric system where the PDMS/carbon black composite is facing a Kapton sheet. Indeed, for the same frequency and acceleration, a net power density of 0.7 $\upmu \mathrm{W}/{\mathrm{cm}}^{2}$ is measured.

The manuscript is organized as follows. First, we remind the general working principle of a capacitive transducer to highlight key optimization parameters. Second, we describe the transducer architectures and the properties of the emulsion based PDMS/carbon black composite. Third, we discuss the results. We then conclude with comparison between the two systems and future ways of improvement.

### Basic considerations on the working principles of the capacitive energy harvester

In contrast to triboelectricity, electrical charges from a capacitive transducer are provided by a DC bias voltage. The change in charge density is directly influenced by the change in capacitance of the transducer due to a mechanical energy input.

#### Capacitive transducer

Capacitance from a capacitive transducer can change in time thanks to a geometrical change of the electrode arrangement (change in electrode distance or surface) but also from a change in the dielectric permittivity (electrostriction), see Table 1.

**Table 1:** The different sources of capacitance variations.

Parameters	Physical origin
$S=S(t)$	Motion/deformation of electrode
$d=d\left(t\right)$	Gap-closing
$\varepsilon =\varepsilon (t)$	Electrostriction/dielectric constant change

$$C\left(t\right)=\frac{{\varepsilon }_{0}\varepsilon S}{d}$$

If we suppose that during a time interval $[{t}_{1};{t}_{2}],$ the capacitance $C\left(t\right)$ is changing from ${C}_{1}$ to ${C}_{2}$, the work $W$ produced by the electrostatic force of the capacitor is given by:

1 $$W={\int }_{{C}_{1}\left({t}_{1}\right)}^{{C}_{2}\left({t}_{2}\right)}\frac{1}{2}{U}_{{C}_{var}}{\left(t\right)}^{2}dC$$

Since ${U}_{{C}_{var}}{\left(t\right)}^{2}$≥ 0, the work $W$ is negative when the capacitance decreases (${C}_{2}\left({t}_{2}\right)<{C}_{1}\left({t}_{1}\right))$. As a result, the energy transfer from the mechanical domain (outside) to electrical domain (inside) occurs when a biased capacitive transducer increases its capacitance.

Using the relation $q(t)=C(t) {U}_{{C}_{var}}\left(t\right)$ and its differential, the work can be rewritten as

2 $$W=\frac{1}{2}{\oint }_{\Gamma }{U}_{{C}_{var}}\left(t\right)dq-q\left(t\right)d{U}_{{C}_{var}}$$

where $\Gamma$ is the path that the variable capacitor follows in the plot (${U}_{{C}_{var}},q\left(t\right)$).

Applying the Green’s theorem to (2) it leads to

3 $$W = - \mathop{{\int\!\!\!\!\!\int}\mkern-21mu \bigcirc} {dqdU_{{C_{{\text{var}}} }} }$$

Finally, $W$ corresponds to the area of the domain by the curve of the plot (${U}_{{C}_{var}},q\left(t\right)$).

The whole point of capacitive transducers is to maximize this area to enhance the mechanical–electrical conversion.

#### Theoretical study of a continuous conditioning circuit

The simplest conditioning circuit demonstrating a generation of electrical power out of variation of a capacitance is the “continuous conditioning circuit”^6^. In this circuit all voltages and currents of the circuit are continuous functions of time.

**Figure 1:**
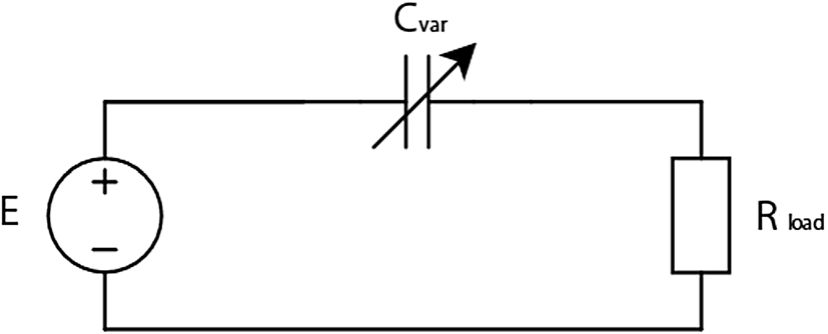
Schematic of the continuous conditioning circuit.

Its design is presented in Fig. 1.

The circuit is composed of a time variable capacitor ${C}_{var} \left(t\right)$, a load resistance ${R}_{load}$ and a DC bias voltage $E$.

The equation describing the electrical behavior of the continuous conditioning circuit is given by Kirchhoff's voltage law:

4 $$E={U}_{{C}_{var}}\left(t\right)+{U}_{{R}_{load}}\left(t\right)={U}_{{C}_{var}}\left(t\right)+{R}_{load }i\left(t\right)$$

The current is written as the derivative of the charge with respect to the time:

5 $$i\left(t\right)=\frac{dq\left(t\right)}{dt}=\frac{d{C}_{var}\left(t\right){U}_{{C}_{var}}}{dt}=C\left(t\right)\frac{d{U}_{{C}_{var}}}{dt}+{U}_{{C}_{var}}\left(t\right)\frac{dC}{dt}$$

When combining (4) and (5) with (6), the differential equation governing the circuit is given by:

6 $$E= {U}_{{C}_{var}}\left(t\right)+{R}_{load} \left[C\left(t\right)\frac{d{U}_{{C}_{var}}}{dt}+{U}_{{C}_{var}}\left(t\right)\frac{dC}{dt}\right]$$

Finally, we obtain the first order following differential equation:

7 $$\frac{d{U}_{{C}_{var}}}{dt}=g\left(t\right)-f\left(t\right) {U}_{{C}_{var}}\left(t\right)$$

with

8 $$f\left(t\right)= \frac{1}{{C\left(t\right)R}_{load}}+\frac{1}{C\left(t\right)}\frac{dC}{dt}$$

9 $$g\left(t\right)=\frac{E}{{C\left(t\right)R}_{load}}$$

#### Numerical simulations

In order to study the operation of the continuous conditioning circuit, we numerically solve, by an Euler explicit scheme, the differential Eq. (7) governing the circuit.

We approximate the time derivative function ${U}_{{C}_{var}}(t)$ by the rate of change of this function over an interval $h$ ($h\to 0$).

10 $$\frac{d{U}_{{C}_{var}}}{dt}=\frac{{U}_{{C}_{var}}\left(t+1\right)-{U}_{{C}_{var}}\left(t\right)}{h}$$

For the study, the capacitive transducer is assumed to move according to the sinusoidal law:

11 $$C\left(t\right)=\frac{{C}_{0}}{k}\mathrm{sin}\left(wt\right)+{C}_{0}$$

where $t$ is the time, $w$ the pulsation ($w=2\pi f$, with $f$ the frequency) and $k$ a positive constant.

We run the simulation for several values of the load resistance and the DC bias voltage. The numerical values of the parameters used in the simulation are given in Table 2.

**Table 2:** Values of fixed and variables parameters used for simulation.

**Fixed parameters**	
$k$	5
${C}_{0}$	200 pF
$f$	30 Hz
$h$	0.0001 s
**Variables parameters**	
$E$	10–100 V
${R}_{load}$	1–50 M$\Omega$

**Figure 2:**
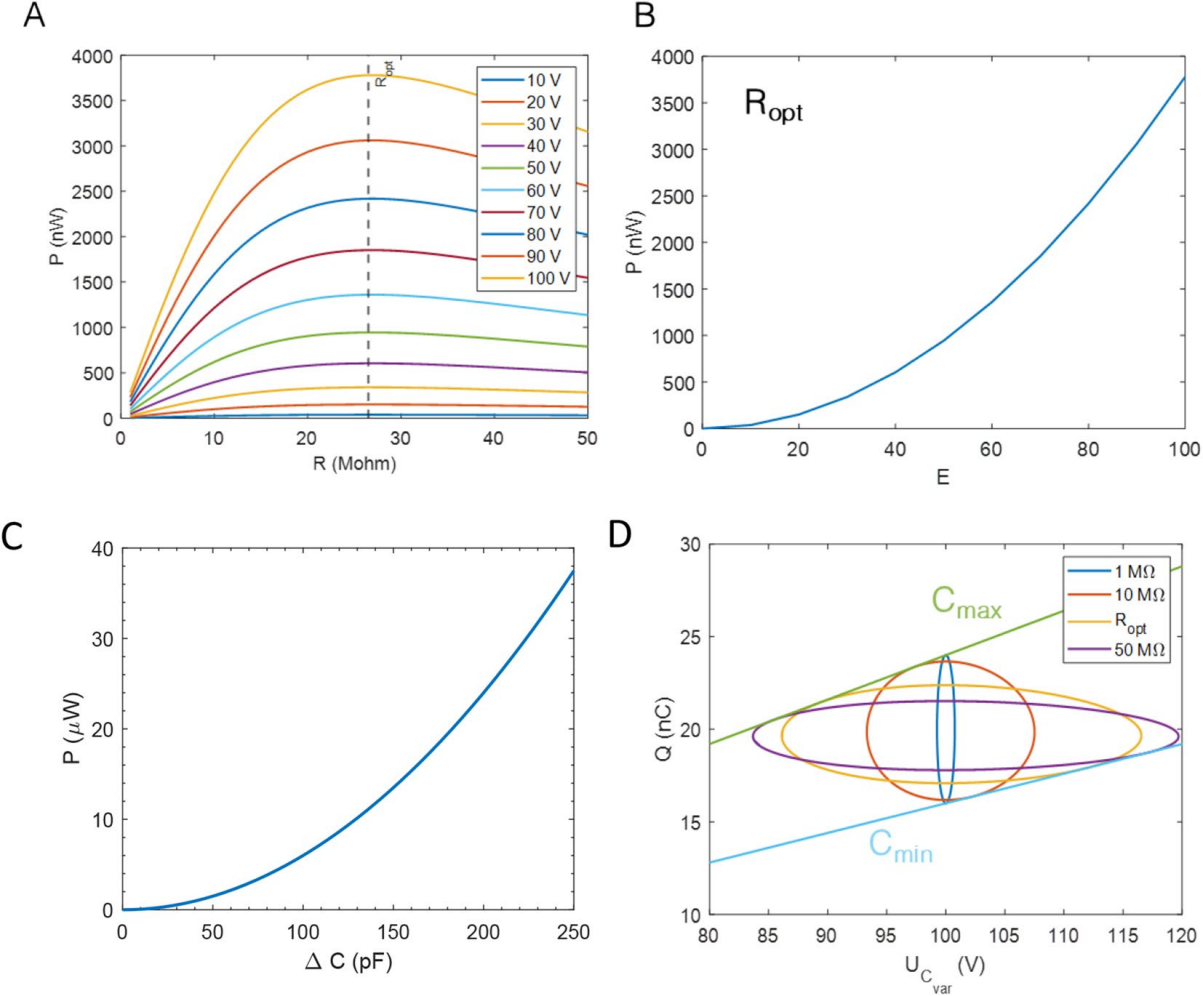
(**A**) Average power $P$ converted to a steady-state by the continuous conditioning circuit for different DC voltage bias E against ${R}_{load}$. The black dashed line highlights the optimal load resistance (**B**) Average power $P$ converted to a steady-state by the continuous conditioning circuit against E (with ${R}_{load}={R}_{opt}$ (**C**) ${(U}_{{C}_{var}},q\left(t\right))$ cycles corresponding to steady-state operation of the continuous conditioning circuit for different load resistances. (**D**) Average power $P$ converted to a steady-state by the continuous conditioning circuit against $\Delta C={C}_{max}-{C}_{min}$, for ${R}_{load}={R}_{optimal}$ and E = 100 V.

Figure 2A presents the plot of the average power generated against the value of the load resistance. This plot is given for different value of bias voltages. The power is maximum when the load resistance is equal to ${R}_{load optimal}= \frac{1}{{C}_{0}w} = 26.5 M\Omega$. Figure 2B shows the average power generated against the DC bias voltage at ${R}_{load optimal}$. The power evolution is quadratic. Figure 2C shows also that the average power generated against $\Delta C={C}_{max}-{C}_{min}$ for a DC bias voltage of 100 V at ${R}_{load optimal}$, follows a quadratic evolution.

In the ${(U}_{{C}_{var}},q\left(t\right))$ plots (Fig. 2D), all possible states are limited by the values of ${Q}_{\mathrm{min}}={C}_{\mathrm{min}}{U}_{{C}_{var}}\left(t\right)={C}_{0}\left(1-\frac{1}{k}\right){U}_{{C}_{var}}\left(t\right)$ and ${Q}_{\mathrm{max}}={C}_{\mathrm{max}}{U}_{{C}_{var}}\left(t\right)= {C}_{0}\left(1+\frac{1}{k}\right){U}_{{C}_{var}}\left(t\right)$. As a function of time, the curves are covered clockwise which means that mechanical energy is converted in to electrical energy thanks to the capacitance variation. The enclosed area is maximal for the optimal load resistance $\frac{1}{{C}_{0}w}$.

### Experiments

**Figure 3:**
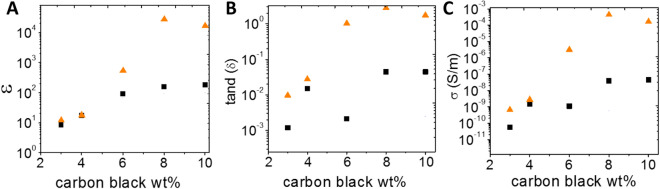
Effective dielectric properties at 100 Hz, 1 V of the CB/PDMS composite (orange triangles), and bilayer composites: PDMS 5 m$\mu$— CB/PDMS composite (black squares).

The soft dielectric layer is a carbon black (CB)/PDMS composite made by using a water-in-oil (W/O) emulsion template (“Materials and methods”). This process provides a fine control of the location of the conductive inclusions which remain confined in the materials pores. The direct carbon dispersion in the PDMS matrix gives a very inhomogeneous dispersion with large aggregates. In the field of dielectric composites, working near and above the percolation threshold allows a huge increase of the materials permittivity. However in theses regimes, the composites suffer from a weakness due to the significant increase of electrical losses^21^ (free charge carriers and dielectric losses). This is a drawback for energy harvesting techniques based on capacitive transducer. In this work, we insert an insulating pure PDMS layer onto the CB/PDMS composite. The insulating layer acts as a blocking layer and prevents discharge of the capacitor. This concept and its theoretical development were recently discussed elsewhere by our research group^20^. So as to identify the optimal carbon concentration to increase the dielectric constant, we investigate the dielectric properties of the CB/PDMS composites at rest with and without PDMS insulating layer (Fig. 3). The CB/PDMS and PDMS layers are respectively 500 $\mu m$ and 5 $\mu m$ thick. These results have already been described by our research group^20^, but we recall the main ones here.

The dielectric composites without insulating layer have a percolation threshold around 5 wt% (Fig. 3A). This carbon concentration separates two areas. Before the percolation, a quasi-constant value of 10 is measured for the dielectric permittivity while the electric conductivity is ${10}^{-9} S {m}^{-1}$. After the percolation, an increase of the dielectric permittivity occurs up to $5.4 \cdot{10}^{2}$ at 6 wt% of carbon black. Meanwhile, the electrical conductivity increases by a factor of ${10}^{2}$, leading to a loss tangent above 1. When an insulating layer is used, the effective dielectric properties of the materials are very affected. Indeed, at carbon black concentrations above the percolation threshold, the PDMS coating limits the electric conductivity under 1—10 $nS {m}^{-1}$. The PDMS coating also conducts to a decrease in the effective dielectric permittivity to $1.8 \cdot {10}^{2}$ for 10 wt% carbon black. The resulting dielectric loss tangent remains very low with $4.5\cdot {10}^{-2}$ for the 10 wt% carbon black formulation, making it well appropriate for its integration into a energy harvesting cantilever beam. Increasing the carbon concentration above 10 wt% makes the emulsion process very complex as the carbon solution goes from a liquid to a solid state.

**Figure 4:**
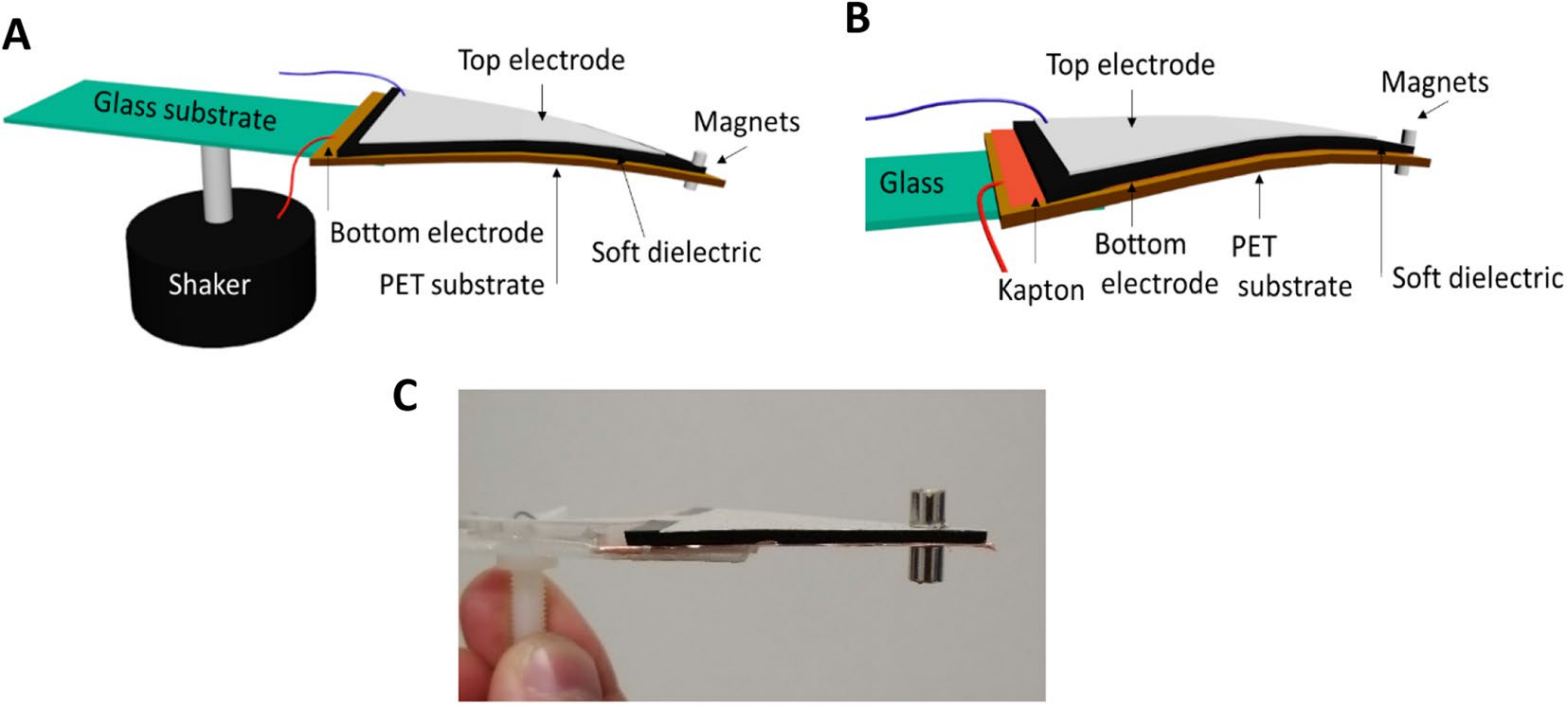
(**A**) Schematic representation of the capacitive energy harvester. (**B**) Schematic representation of the triboelectric energy harvester. (**C**) Photograph of the capacitive energy harvester.

For the next steps, the 10 wt% CB/PDMS materials with the PDMS insulating layer are chosen to be integrated into the cantilever beams as they showed the highest dielectric constant. The architectures of the cantilever beams under investigation are illustrated in Fig. 4. The detailed fabrication is described in the Materials and methods part. For both harvesters, we decided to work with a triangular cantilever with a seismic mass as this shape provides the highest deformation compared to the rectangular or trapezoidal shapes^22^. The capacitive harvester involves the CB/PDMS composite (soft dielectric) and a copper electrode (bottom electrode) as two surfaces facing each other while the triboelectric harvester involves a CB/PDMS composite and a Kapton/copper layer.

**Figure 5:**
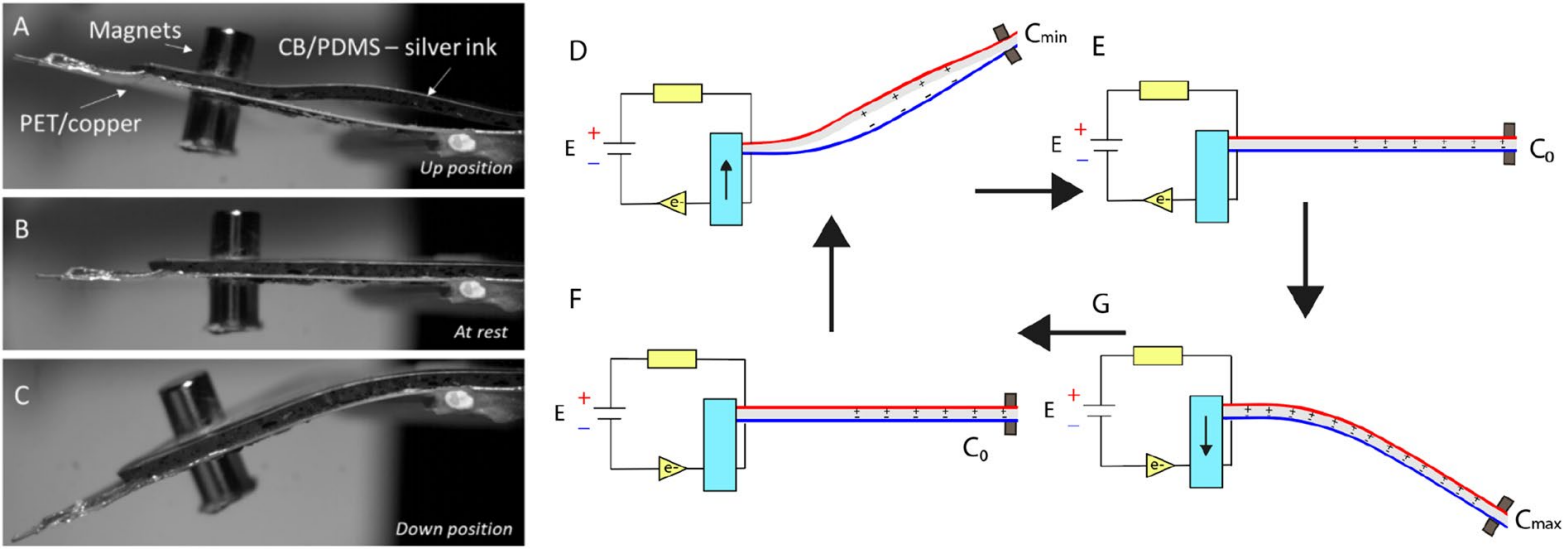
(**A**) Photograph of the capacitive harvester in up position. (**B**) Photograph of the capacitive harvester at rest. (**C**) Photograph of the capacitive harvester in down position. (**D–G**) Working principle of a capacitive transducer in a cantilever beam configuration (red and blue layers represents the two electrodes connected to the DC bias **E**).

For the two systems, the two bases of the triangular surfaces are glued together while the tips are simply fixed together by a magnet. As shown in the Video 1 (Video_1_capacitive_havester, Supporting Information) the magnets are very efficient to fix the two extremities when the capacitive harvester is in action. Thus, when the beam is vibrated, the two layers are free to bond or detach (see Fig. 5). For the capacitive harvester this freedom of movement allows the capacitor made by the top/bottom electrodes and the soft dielectric to change its capacitance (by changing the contact surface, electrodes distance and dielectric material—air to material).

When the cantilever is up, the top layer is detaching from the bottom layer, the capacitance is at this moment minimal as the dielectric is changing from CB/PDMS to air, the distance is increasing and the contact surface is decreasing (Fig. 5D). The capacitor accepts fewer charges coming from the DC bias and as a consequence a current is circulating. Conversely, when the cantilever is in down position the capacitance is higher, as the gap between the top and bottom electrodes is decreasing. As more charges are accepted by the capacitor, a reverse current is induced (Fig. 5G). Finally, the cantilever’s oscillations provide an alternating current resulting from the mechanical to electrical conversion.

**Figure 6:**
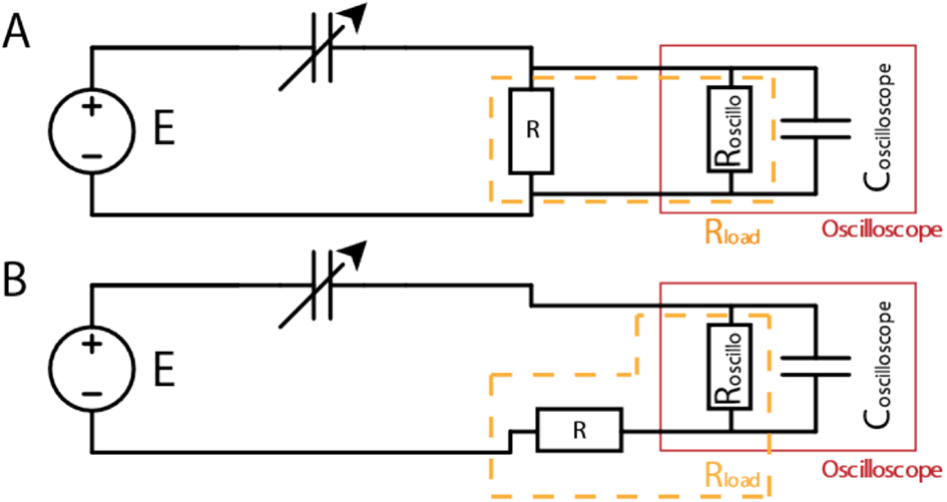
${R}_{load}$ values for two oscilloscope configurations. (**A**) The oscilloscope is in parallel with respect to $R$, thus ${R}_{load}=\frac{R {R}_{oscilloscope}}{R+ {R}_{oscilloscope}}$. (**B**) The oscilloscope is in series with respect to $R$, thus ${R}_{load}={R}_{oscilloscope}+R$.

For the measurements, the capacitive harvester is connected to a resistance $R$ and a DC bias voltage $E$ (several 9 V alkaline batteries) to continuously condition the circuit. An oscilloscope (Keysight InfiniiVision 1000 X) measures the voltage ${U}_{oscillo}(t)$ at the internal resistance of the oscilloscope (${R}_{oscilloscope}=10\mathrm{ M\Omega }$). The internal load resistance contributes to the total load resistance ${R}_{load}$ depending on how the oscilloscope is connected to the circuit (Fig. 6).

As a result, to get ${R}_{load}>{R}_{oscilloscope}$, the oscilloscope will be connected in series with respect to $R$ and ${U}_{Rload}\left(t\right)=\frac{{R}_{load}{U}_{oscillo}\left(t\right)}{{R}_{oscilloscope}}$ although for ${R}_{load}<{R}_{oscilloscope}$, the oscilloscope will be connected in parallel with respect to $R$ and ${U}_{Rload}\left(t\right)= {U}_{oscillo}\left(t\right).$

The cantilever is subjected to vibrations using a shaker (4810 Bruel & kjaer) which drives it into different mechanical frequencies from 1 to 50 Hz. An accelerometer (IEPE Bruel & Kjaer, 10 mV/g,) is fixed on the cantilever to measure the shaker acceleration.

It is worth mentioning that ${U}_{Rload}(t)$ contents several contributions (parasitic and harmonics) in addition to the main one which is at the mechanical frequency of excitation of the beam. Thus, we choose to analyse the electrical signal with a numerical FFT (Fast Fourier Transform), to keep the rms (root mean square) contribution from the mechanical excitation frequency (${U}_{R(\mathrm{FFT},rms)}({\omega }_{shaker})$). The average harvested power per cycle $\stackrel{-}{{P}_{total}}$ is calculated using Eq. (12). We develop in the Supporting Information section (see Fig S1) one example of this calculation.

12 $$\stackrel{-}{{P}_{total}}=\frac{1}{\uptau }{\int }_{0}^{\tau }\frac{{U}_{Rload}{\left(t\right)}^{2}}{{R}_{load}}=\frac{{U}_{R(\mathrm{FFT}, rms)}{\left({\omega }_{shaker}\right)}^{2}}{{R}_{load}}$$

where ${\omega }_{shaker}$ is the shaker pulsation.

**Figure 7:**
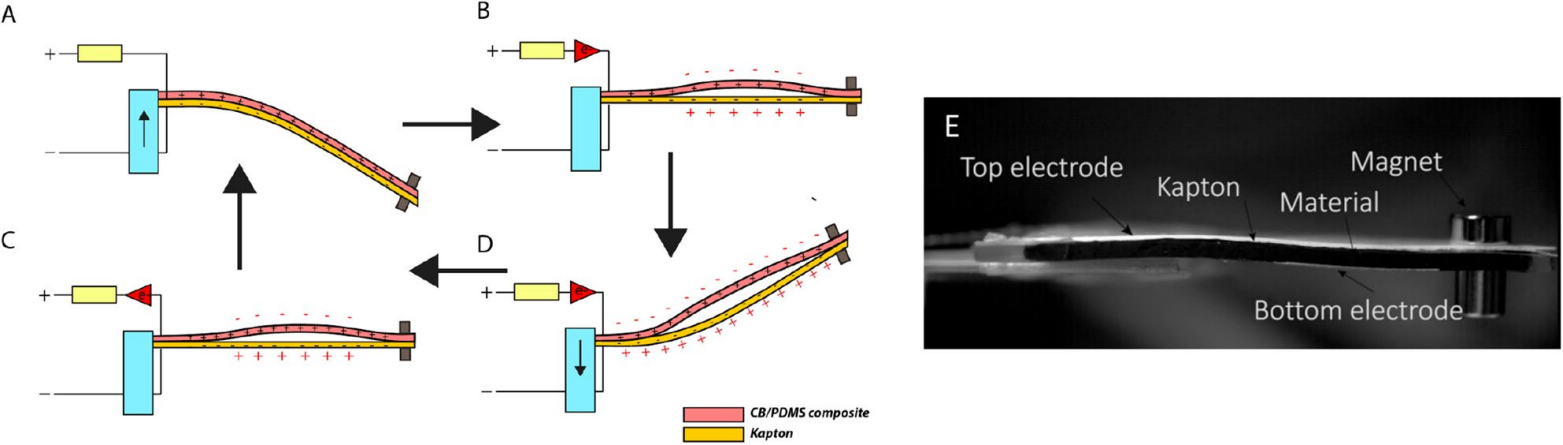
(**A**–**D**) Working principle of the triboelectric transducer in a cantilever beam configuration. (**E**) Photograph of the triboelectric harvester at rest.

For the triboelectric harvester, the freedom of movement of the top electrode allows the charges generation in a contact separation mode between the Kapton and CB/PDMS layers. At first, the shaker introduced displacement of the cantilever to its down position, the two dielectrics (CB/PDMS and Kapton) are brought into contact with each other (Fig. 7A). Video 2 from the Supporting Information section shows the triboelectric harvester in action and Fig. 7E a photograph of the device. The triboelectric effect occurs when surface charges transfer at the contact area. According to the triboelectric series^23^, the Kapton has a tendency to be negatively charged. We will consider that the composite CB/PDMS with be positively charged. Once the two polymers separate (Fig. 7B,D), a potential difference is then established between the two electrodes since the opposite triboelectric charges are separated. As the two electrodes are shorted, the established potential difference drives electrons from the bottom electrode to the top electrode.

Once the cantilever is down (Fig. 7C), reduction of the interlayer distance makes the electric potential between the two electrodes decreasing. As a consequence, electrons are driven from the top electrode back to the bottom electrode, reducing the amount of induced charges. A reverse current is thus generated. When the Kapton and CB/PDMS composites are in contact again, all induced charges are neutralized (Fig. 7A).

To make the measurements, the triboelectric harvester is connected to a resistance $R$. The same, previously described, oscilloscope connections are used depending on the ${R}_{load}$ we wanted to get.

## Results and discussion

We note that in the following text, the term power refers to average power and not to instantaneous power or peak power as may be done in some works dealing with triboelectric studies. The peak power will be reported under the notation ${P}_{peak}.$

### Capacitive harvester

The total harvested power ${P}_{total}$ is determined by measuring the voltage at the load resistance ${R}_{load}$ when the cantilever is mechanically excited using a shaker as depicted in Fig. 4A.

**Figure 8:**
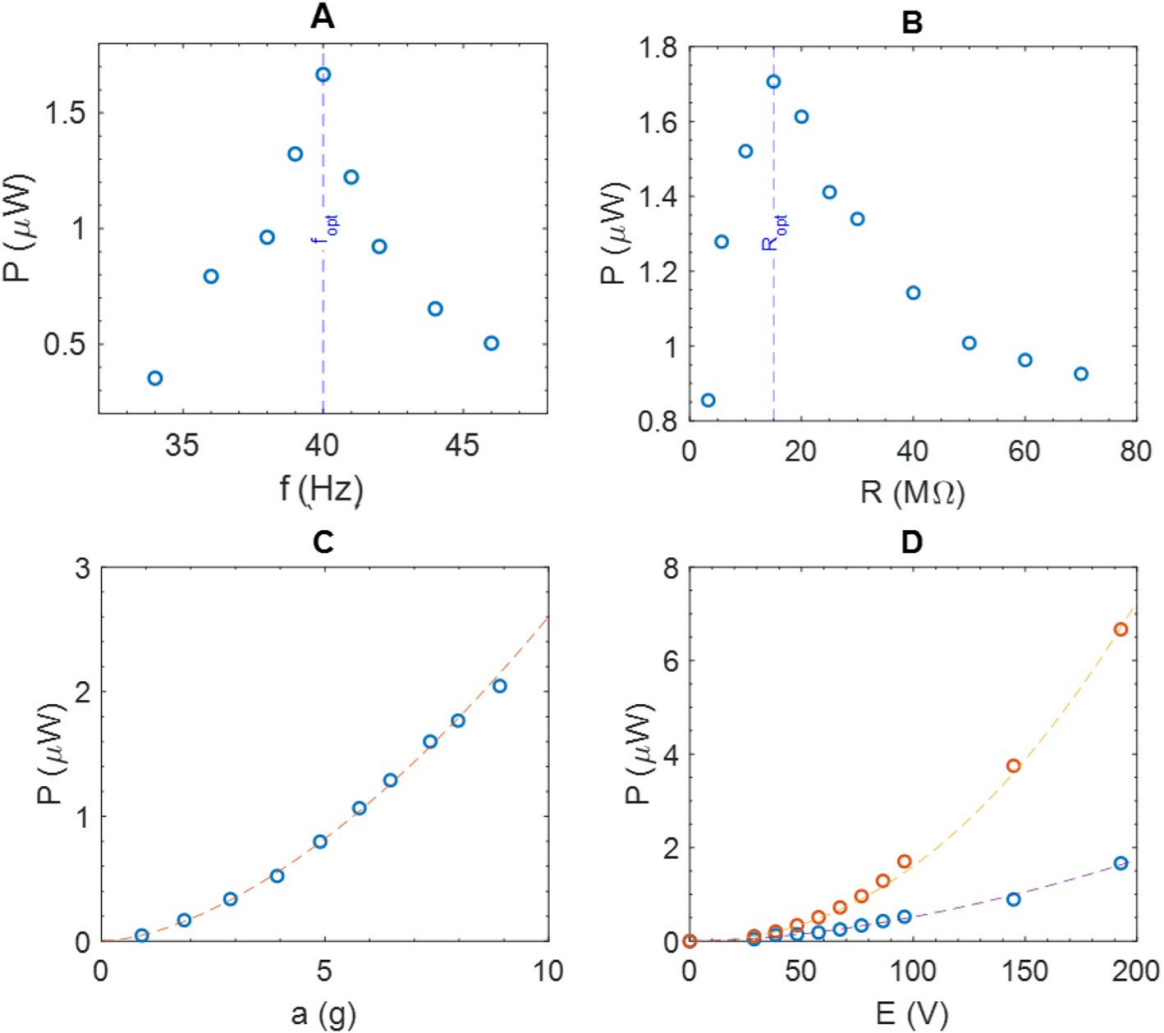
(**A**) Power harvested as a function of the cantilever excitation frequency for a = 7.3 g, ${R}_{load}$=15 $\mathrm{M\Omega }$, $E=96\mathrm{V}$. The resonant frequency ${f}_{opt}$(40 Hz) is highlighted by a blue dashed line. (**B)** Power harvested as a function of the load resistance (${R}_{load}$) for a = 7.3 g, ${f}_{opt}$ and $E=96\mathrm{V}$. The optimal load resistance ${R}_{opt}$ is highlighted by a blue dashed line. (**C**) Power harvested as a function of the acceleration amplitude, for the resonant frequency ${f}_{opt}$, ${R}_{load}$=15 $\mathrm{M\Omega }$, $E=96\mathrm{V}$. (**D**) Power harvested as a function of the bias voltage $E$ for a = 3.9 g (blue circles) and a = 7.3*g* (orange circles), at ${R}_{load}$,${f}_{opt}.$

The harvested power as a function of the frequency of the mechanical excitation is presented in Fig. 8A. The load resistance is fixed at $${R}_{load}$$=15 $$\mathrm{M\Omega }$$ , $$E=96 V$$ and a peak acceleration of 7.3*g* (71.6 $$m{s}^{-2}$$) is applied by the shaker. The output power is maximized for a frequency of 40 Hz which corresponds to the fundamental natural resonant frequency ($${f}_{opt})$$ of the cantilever.

A power of 1.8 $\upmu \mathrm{W}$ is generated at this frequency although a decrease of power of 44% was observed for an offset of 2 Hz from the resonant frequency. Also, the output power depends on the load resistance ${R}_{load}$, which has to be optimized to recover the maximum power. Optimization is established by varying the load resistance and illustrated in Fig. 8B. Successive measurements are performed with different values of ${R}_{load}$ varying from 3.3 $\mathrm{M\Omega }$ to 70 $\mathrm{M\Omega }$. The maximum power is measured with ${R}_{load}$=15 $\mathrm{M\Omega }$ using a peak acceleration of 7.3*g* at the resonant frequency.

Taking into account the surface (7.5 cm^2^) of the capacitive transducer, it leads to a maximum raw power density of 0.24 $\upmu \mathrm{W}/{\mathrm{cm}}^{2}$ at the resonant frequency, the optimal load resistance, an acceleration of 7.3*g* and a bias voltage of 96 V. For lower acceleration amplitudes, the harvested power is decreasing following a quadratic power law (exponent close to 2)$: P=5.7\cdot {10}^{-2}{ a}^{1.7} \upmu {\mathrm{W}}$ ($a$ is the acceleration amplitude, in g) as shown by the orange dashed plot in Fig. 8C. As predicted by the theoretical analysis of the circuit (Fig. 2B), the bias voltage $E$, has also a quadratic influence on the harvested power (exponent close to 2). Figure 8D shows the evolution of the harvested power as a function of the applied bias voltage. For two different accelerations (a = 3.9*g* and a = 7.3*g*), the power laws are respectively ${P}_{a=3.9 g}=1.6 \cdot{10}^{-4}{ E}^{1.7}\upmu {\mathrm{W}}$ (E in V) and ${P}_{a=7.3 g}=7\cdot {10}^{-5}{E}^{2.2}\upmu {\mathrm{W}}$ (E in V).

**Figure 9:**
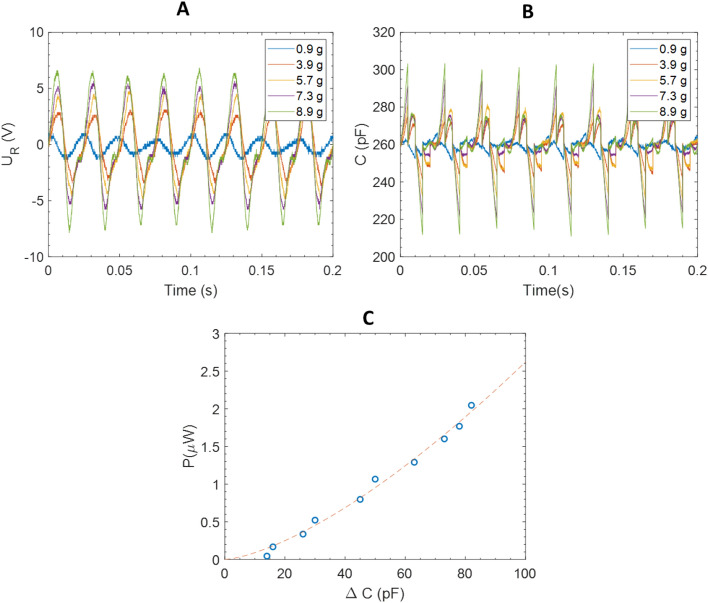
(**A**) Load resistance voltages as a function of the time for different accelerations (${f}_{0}, {R}_{opt}, E=96 V$) (**B**) Calculated capacitances as a function of the time for different accelerations (${f}_{0}, {R}_{opt}, {C}_{0}, E=96 V$) (**C**) Power harvested as a function of $\Delta C={C}_{max}-{C}_{min}$.

It should be noted that in the absence of polarization, the power recovered in this system is zero (Fig. 8D). This means that the mechanisms measured here are of capacitive origin and not triboelectric. Based on the theoretical predictions previously described (Fig. 2A), the optimal resistance is given by ${R}_{load optimal}= \frac{1}{{C}_{0}w}$. With $w=2\pi {f}_{0}$, ${f}_{0}=40 Hz$ and ${R}_{load optimal}=$ 15 $\mathrm{M\Omega }$, ${C}_{0}$ is estimated at 265 pF. This value is confirmed experimentally, by the measure of the capacitance at rest ${C}_{0}$ at ${f}_{AC}$ =40 Hz. The evolution of the capacitance as a function of the time $C\left(t\right)$, can be numerically solved by using the differential Eq. (7) governing the circuit, ${U}_{Rload}\left(t\right)$ measurements, and the initial condition $C(0)={C}_{0}$ (${C}_{0}$ is used as initial value for each period). Figure 9B shows the calculated capacitances as a function of time from experimental value of ${U}_{Rload}$ (Fig. 9A). It can be seen that increasing the acceleration of the cantilever increases the capacitance variations. Consequently, the harvested power increases by a quadratic power law (P = $3.2 \cdot {10}^{-3}{\Delta C}^{1.5} \upmu {\mathrm{W}} (\Delta C {\text{ in pF}})$ (Fig. 9C). This evolution was previously predicted (Fig. 2C).

Now we expose the calculations of the loss power for the capacitive harvester. When the harvester is subjected to a bias voltage ${V}_{DC}$, a continuous voltage is measured at the load resistance ${R}_{load}$ corresponding to a resistive loss from the capacitive harvester (internal leak resistance).To extract the DC contribution of the voltage at the load resistance, we choose to analyse the electrical signal with a numerical FFT, to keep the rms (root mean square) contribution at 0 Hz (${U}_{R\left(FFT, rms\right)}(0Hz)).$

Thus, the power loss is calculated using EQ. 13.

13 $${P}_{loss} =\frac{{U}_{R(FFT, rms)}{\left(0 Hz\right)}^{2}}{{R}_{load}}$$

Table 3, presents the calculated losses for $f$=40 Hz, ${R}_{load}$=15 $\mathrm{M\Omega }$, a = 7.3 g and different values of voltage bias ${V}_{DC}$.

**Table 3:** ${P}_{loss}$ and ${P}_{harvested}$ regarding the applied bias voltage.

${V}_{DC}$(V)	${P}_{loss}$(p $W$)	${P}_{harvested}$($\upmu \mathrm{W}$)
58	1.0	0.5
96	4.1	1.7
145	6.3	3.8
193	9.2	6.7

Table 3 shows even if losses increase with the applied bias voltage, their values are quite negligible compared to the harvested power.

### Triboelectric harvester

For comparison, the performances of the triboelectric harvester using the same composite CB/PDMS, contact area, tip magnets and excitation frequency are investigated. The main difference between the previously described harvester is the absence of bias voltage $E$ and the presence of a Kapton layer on the bottom electrode of the cantilever (see Fig. 4B).

**Figure 10:**
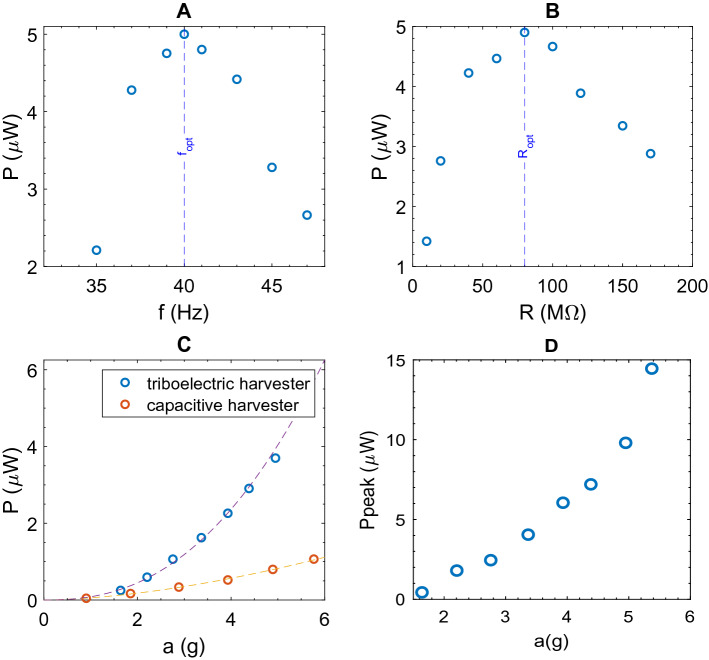
(**A**) Power harvested as a function of the cantilever excitation frequency for a = 5.4 g, ${R}_{opt}$=80 $\mathrm{M\Omega }$, no bias. The resonant frequency ${f}_{opt}$(40 Hz) is highlighted by a blue dashed line. (**B**) Power harvested as a function of the load resistance (${R}_{load}$) for a = 5.4 g, ${f}_{opt}.$ The optimal load resistance ${R}_{opt}$ is highlighted by a blue dashed line. (**C**) Power harvested as a function of the acceleration amplitude (blue circles), for the resonant frequency ${f}_{opt}$, ${R}_{opt}$=80 $\mathrm{M\Omega }$. Comparison with the capacitive harvester (orange circles, E = 96 V). (**D**) Pick power as a function of the acceleration amplitude (blue circles), for the resonant frequency ${f}_{opt}$, ${R}_{opt}$=80 $\mathrm{M\Omega }$.

Figure 10B shows the power harvested as a function of excitation frequency. At the resonant frequency of 40 Hz, a harvested power of 5 $\upmu \mathrm{W}$ for an acceleration of 5.4*g* is measured (${R}_{load}$=80 $\mathrm{M\Omega }$). For the same acceleration, we remind that the capacitive transducer harvested 1.0 $\upmu \mathrm{W}$, with a bias voltage of 96 V (see Fig. 8C). The load resistance optimization is illustrated in Fig. 10A. By contrast to the capacitive transducer study the optimal resistance is much higher with a value of 80 $\mathrm{M\Omega }$. An increase ($P=8.5 \cdot {10}^{-2} {a}^{2.4} \upmu {\mathrm{W}}$ ($a$ is the acceleration amplitude, in g) of the recovering power with the acceleration of the beam is determined experimentally, as shown in Fig. 10C (purple dashed line). As illustrated in the same figure, the harvesting power is much higher with the triboelectric system than for the capacitive one.

Comparatively, for the triboelectric system, under an acceleration of 3.9*g*, the cantilever harvests 2.2 $\upmu \mathrm{W}$. So as to get the same recovery power under the same acceleration and by using the power fit, previously described, a DC bias voltage of 274 V is required ($E={\left(\frac{2.2}{1.6*{10}^{-4}}\right)}^{1.7}=274 {\text {V}}$), for the capacitive harvester.

We specify that in the previous paragraph only average powers have been measured. The measurement of the instantaneous power ${P}_{peak}$ of the system is presented Fig. 10D, as a function of the acceleration of the beam. Thus, at 40 Hz, an instantaneous harvested power of 14.4 $\upmu \mathrm{W}$ for an acceleration of 5.4 g is measured (${R}_{load}$=80 $\mathrm{M\Omega }$).

### Influence of humidity on both recovery systems

**Figure 11:**
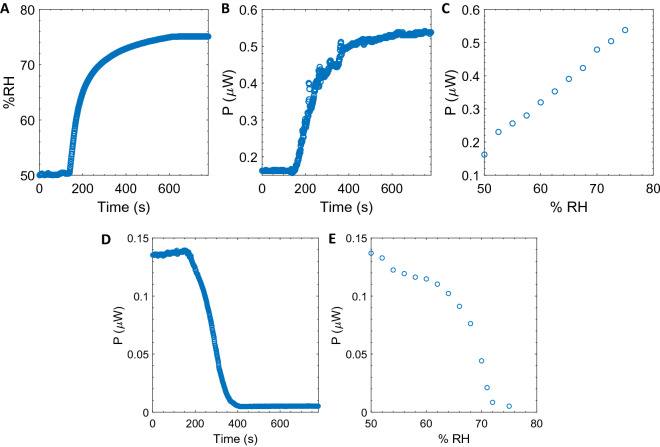
Capacitive harvester: (**A**) Relative humidity inside the jar as a function of the time. (**B**) Harvested power as a function of the time (**C**) Harvested power as a function of the relative humidity. Triboelectric harvester: (**D**) Harvested power as a function of the time (**E**) Harvested power as a function of the relative humidity.

To go further in the comparison of the two systems, we studied the influence of humidity on the recovery performance of capacitive and triboelectric beams. For this purpose, we operated the harvesters in a glass bell jar equipped with a water tank and where the humidity was measured at the same time as the electrical signals (see Materials and methods section for the experiment description). At the beginning of the experiment the bell jar is not closed and the air inside is identical to the surrounding air (50% of relative humidity (RH)), see Fig. 11A. The capacitive harvester operates at T = 25 °C with a voltage of 96 V, a frequency of 40 Hz, a load resistance of 15 $M\Omega$ and an acceleration of 1.9*g*. Under these conditions, the harvester recovers: 0.16 $\upmu \mathrm{W}$. After 180 s, the jar is hermetically sealed, and the water in the tank begins to evaporate. The relative humidity of the bell reaches equilibrium after 500 s at 75% RH (see Fig. 11A).

Figure 11B shows that the recovered power follows the same evolution over time as the relative humidity. A linear evolution of the power is measured as a function of the humidity of the jar (Fig. 11C). At 75% of RH, the harvesting power is increased by almost a factor 3. This increase in power is probably due to a change in the dielectric between the two electrodes of the beam from air-PDMS/CB to air/water-PDMS/CB. Since water has a higher dielectric permittivity than air (78.5 compared to 1), the recovered power increases. The same experiment is performed with the triboelectric harvester (T = 25 °C, $f$=40 Hz, ${R}_{load}$=80 $M\Omega$, acceleration of 1.9*g*). Compared to the capacitive harvester, humidity is unfavourable to the triboelectric one. As shown in Fig. 11D,E, there is a clear decrease in the power recovered from $0.14$ to $4 \cdot{10}^{-3}\upmu {\mathrm{W}}$ when the humidity changes from 50 to 75%. Even if the origin of this decrease is out the scope of our study, this experiment suggests that a high humidity dissipates electrical charges as water molecules make the surfaces more conductive^9^ or that changes in friction coefficient of the materials occur making the contact between them less favourable^24^. It should be noted, however, that it seems possible to encapsulate the triboelectric device in PDMS to make it non-sensitive to moisture.

## Conclusion

We presented a new cantilever resonator, specifically developed for real vibrational energy harvesting. Our composite CB/PDMS was associated with a thin insulating layer of pure PDMS to limit electrical losses. We succeeded in evaluating the energy scavenging abilities of this cantilever in two transducers modes: capacitive and triboelectric transducers. We recall that in this work the term power refers to averaged power and not instantaneous power or peak power as it can be done in some work dealing with triboelectric studies. At the resonant frequency of 40 Hz a recovery power of 0.3 $\upmu \mathrm{W}/{\mathrm{cm}}^{2}$ for an acceleration of 3.9*g* is measured in triboelectric configuration (instantaneous power density of 1.9 $\upmu \mathrm{W}/{\mathrm{cm}}^{2}$). The same harvesting performances are measured in the capacitive transducer, for identical frequency and acceleration, by using a 0.15 $\mathrm{V}\;\upmu {\mathrm{m}}^{-1}$ bias. We are aware that this system does not achieve the best performance of today's triboelectric harvesters^25,26^, nevertheless the comparison between two systems using the same material but different operating principles remains valuable. The main advantage of the triboelectric transducer is the absence of bias to polarize the material, which makes it possible, on the one hand, to limit the total size of the harvester and, on the other hand, to reduce its weight. Nevertheless, it has been shown^9,24^ that maintaining a constant charges density is challenging because triboelectric charges are dependent on humidity and pressure conditions. The large decrease measured in the experiment we conduct, confirms this effect. This is not the case for the capacitive harvester where moisture is advantageous to increase the harvesting performance as the dielectric constant of wet air is higher than dry air. Thus, triboelectric devices may require packaging to operate in varying environment, in particular for harvesting ocean wave energy where moisture is high. In view of these delicate operating conditions, the capacitive harvester appears to be easier to optimize because the theoretical understanding is more complete. For example, an increase in the charge density of the system can be easily achieved because, unlike triboelectricity, the charges are provided by an external polarization. Thus, we have shown that working at 40 Hz, 3.9*g* and a bias of 0.30 $\mathrm{V}\;\upmu {\mathrm{m}}^{-1}$, a power density of 1 $\upmu \mathrm{W}/{\mathrm{cm}}^{2}$ is harvestable. In summary, the triboelectric harvester appears more suitable for powering small devices, so the space requirement must be limited and therefore the environmental conditions are known^27^. On the other hand, the capacitive harvester appears more suitable for recovery on large structures where pressure and humidity conditions can fluctuate (wave energy recovery^28^ for example) and where the size of the harvester (presence of a polarization battery) is not important. Nevertheless, even if humidity is not a problem for the performance of the capacitive harvester, the presence of liquid water, especially salty water, may be the cause of a short circuit between the electrodes or corrosion of the cables. To prevent these phenomena, an encapsulation of the structure will be necessary.

Further improvements of the presented systems lie in the design of the capacitive cantilever. We believe that optimization of the layers thickness could increase the power density by increasing the strain of the electroactive material. Indeed, the change of capacitance, a key parameter for increasing the harvested energy, is dominated by the applied strain. Moreover as mechanical vibrations are broadly dispersed, a larger resonant frequency bandwidth can be simply done by a parallel arrangement of single beam structures using different seismic masses^29^, in particular to match very low frequencies (0.1 to 10 Hz, human motion). In addition, the resonant variable capacitor cantilever could find applications for low-cost frequency or accelerator sensors in vibrating structures.

## Materials and methods

### CB/PDMS composites formulation

This process has already been described by our research group^19,20^. We first prepare an emulsion and disperse a solution of carbon black particles in water in a mixture of PDMS (RTV615 Momentive), and curing agent (RTV615 Momentive, 10% in weight with respect to the PDMS phase). To ensure the stability of the emulsion we use lauryl PEG-8 dimethicone (Silube J208-812, Siltech, 5.0 wt% of the final mixture) as a surfactant. The exact composition of the water phase is 5*g* of Arabic gum (Sigma Aldrich) for 95*g* of deionized water. To homogeneously disperse the desired amount of carbon black powder (Alfa Aesar) we use a sonicator (for 1 h at 400 W). We progressively add the carbon black dispersion to the oil phase under manual stirring up to a water:oil mass ratio of 80%wt to emulsify the system. The water-in-oil emulsion loaded with carbon black particles is spread with a stencil into a circular plastic mold. The diameter of the mold is 24 mm and its depth 125 microns. We set a second plastic surface on the emulsion to confine this latter between two flat surfaces. The PDMS polymer is cured without evaporation in a warm water bowl (90 °C) for 4 h. The relative humidity in these conditions is 100%. Then, the solid material layer is removed from the two plastic surfaces and dried in an oven for 1 h at 150 °C. As PDMS is permeable to water vapor, droplets containing carbon black particles dry. At the end of the process we obtain a foam structure with spherical-shaped pores covered by carbon black particles.

### Dielectric properties measurements

We measure the electrical conductivity and dielectric permittivity of the samples between two metallic disc electrodes at a frequency of 100 Hz under an applied voltage of 1 V using an impedance analyzer (Bio-Logic Impedance Analyser, MTZ-35). A calibration procedure removing the contribution of the polarization of the electrodes is used to determine the dielectric permittivity and the conductivity of the sample as a function of frequency^30^. All the experiments are performed at room temperature.

### Cantilevers fabrication

#### Capacitive harvester

A 7.5 cm^2^ triangular piece of 125 m$\upmu$ thick polyethylene terephtalate (PET-Mylar, Radiospare) is cut. This PET substrate acts as a supporting layer for the cantilever. Copper adhesive (Radiospare) tape is used on the PET layer as the bottom electrode of the cantilever. Then a triangular piece of porous CB/PDMS composite with an isolating layer of 5 microns of PDMS (7.5 cm^2^ × 1.8 mm) is cut. A silver conductive ink is coated on the composite to get the top electrode. The coating is protected by a thin layer of PDMS. Finally, the two triangular parts (PET and composite) are maintained contiguous: the triangular base of the two parts are glued together while the two tips are stick by two magnets (0.35*g* each). Figure 4 shows the final structure of the cantilever. The cantilever is glued to a glass slide, which acts as a support for the shaker. The electrical contacts are established by bonding conductive wires to both electrodes using pieces of conductive copper tape (Radiospare).

#### Triboelectric harvester

The structure of the triboelectric harvester is very similar to the capacitive harvester. Surface and thickness are same. The main difference is the presence of a Kapton sheet (100 $\upmu m$ of thickness, Radiospare) on the bottom electrode. Copper adhesive (Radiospare) tape is used under the Kapton layer as the bottom electrode of the cantilever. The Kapton/copper layer is glued on a PET substate by using double-sided tape.

### Moisture influence measurements

The recovery device as well as the vibrating pot are placed in a hermetic bell (Kartell) where a reservoir of water had been placed. This bell is equipped with a sensor of humidity and temperature (Thorlabs, acquisition frequency of 1 Hz) and allowed the passage of the various cables of the electric circuit. At the beginning of the experiment the bell is not closed and the air inside is identical to the surrounding air (50% of relative humidity (RH)). Then the bell is hermetically sealed, and the water in the tank begins to evaporate. While the water is evaporating, the humidity and harvesting performances are both recorded. The oscilloscope continuously acquires the voltage across the load resistance. A FFT (Fast Fourier transform) is performed directly by the oscilloscope on a 2-s signal. The maximum peak value of the FFT (${U}_{RMS}$) at the studied frequency is recorded. The recovered power is then calculated by using Eq. (12).

## Supplementary information

Supplementary Information.

Supplementary Video 1.

Supplementary Video 2.
