# Supplementary material for: Integration of a soft dielectric composite into a cantilever beam for mechanical energy harvesting, comparison between capacitive and triboelectric transducers
Source: Sci Rep. 2020 Nov 26;10:20681. doi: 10.1038/s41598-020-77581-2 (PMC7692552; doi:10.1038/s41598-020-77581-2)
Supplement: Supplementary file 1 — Supplementary Information. [file 41598_2020_77581_MOESM1_ESM.docx]

Integration of a soft dielectric composite into a cantilever beam for mechanical energy harvesting, comparison between capacitive and triboelectric transducers.

Mickaël Pruvost ^a*^, Wilbert J. Smit ^a^, Cécile Monteux ^b^, Pablo Del Corro^c^, Isabelle Dufour^c^, Cédric Ayela^c^, Philippe Poulin ^d^, Annie Colin ^a^

a MIE team, Chimie Biologie et Innovation, ESPCI Paris, PSL University,
CNRS, 75005 Paris, France

b Sciences et Ingénierie de la Matière Molle, ESPCI Paris, PSL University,
CNRS, Sorbonne Université, 75005 Paris, France

c Laboratoire IMS, CNRS, Université de Bordeaux, 33600 Pessac, France

d Centre de Recherche Paul Pascal, CNRS, Université de Bordeaux, 33600 Pessac, France

* Corresponding author. E-mail adress: [mickael.pruvost@espci.fr](mailto:mickael.pruvost@espci.fr)

**Supporting Information**

- *Precisions on how the harvested power is calculated:*

First, we record the voltage at the load resistance $R_{load}$ (FIG. S1 A) and then we apply a FFT (Fast Fourier Transform) to the voltage signal (FIG. S1 B). From the FFT signal (V, RMS), we extract the maximum value of the amplitude at the working frequency f. Finally, by using equation (12) from the manuscript ($\bar{P_{total}}=\frac{U_{R(FFT, rms)}\left( \omega_{shaker} \right)^{2}}{R_{load}})$, we calculate the harvested power.

In this example:

f=34 Hz, R= 15$M\Omega$, $U_{R(FFT, rms)}$= 2.33 V so $\bar{P_{total}}=\frac{{2.33}^{2}}{15 {10}^{6}}=0.35 \mu W$

**A**

**B**

FIG. S1: A) Voltage at the load resistance as a function of the time. FFT amplitude of the voltage at the load resistance (50 Hz pic is due to electrical noise from the French electrical network)
